# A Microbial Feed Additive Abates Intestinal Inflammation in Atlantic Salmon

**DOI:** 10.3389/fimmu.2015.00409

**Published:** 2015-08-19

**Authors:** Ghana Vasanth, Viswanath Kiron, Amod Kulkarni, Dalia Dahle, Jep Lokesh, Yoichiro Kitani

**Affiliations:** ^1^Faculty of Biosciences and Aquaculture, University of Nordland, Bodø, Norway

**Keywords:** Atlantic salmon, microbial feed additive, *Pediococcus acidilactici*, probiotic, intestinal inflammation, histology, proteins, genes

## Abstract

The efficacy of a microbial feed additive (Bactocell^®^) in countering intestinal inflammation in Atlantic salmon was examined in this study. Fish were fed either the additive-coated feed (probiotic) or feed without it (control). After an initial 3-week feeding, an inflammatory condition was induced by anally intubating all the fish with oxazolone. The fish were offered the feeds for 3 more weeks. Distal intestine from the groups was obtained at 4 h, 24 h, and 3 weeks, after oxazolone treatment. Inflammatory responses were prominent in both groups at 24 h, documented by changes in intestinal micromorphology, expression of inflammation-related genes, and intestinal proteome. The control group was characterized by edema, widening of intestinal villi and lamina propria, infiltration of granulocytes and lymphocytes, and higher expression of genes related to inflammatory responses, *mul1b*, *il1b*, *tnfa*, *ifng*, compared to the probiotic group or other time points of the control group. Further, the protein expression in the probiotic group at 24 h after inducing inflammation revealed five differentially regulated proteins – Calr, Psma5, Trp1, Ctsb, and Naga. At 3 weeks after intubation, the inflammatory responses subsided in the probiotic group. The findings provide evidence that the microbial additive contributes to intestinal homeostasis in Atlantic salmon.

## Introduction

Feed ingredients of plant origin have become integral parts of aquafeeds of even carnivorous fish species such as Atlantic salmon. Soybean-induced enteritis in farmed fish, which has similarities to intestinal enteropathies in human beings (1), has been acknowledged as a major nutritional pathology. Soybean contains saponins that cause distal intestinal inflammation (2, 3), which is characterized by intestinal fold height shortening, enterocyte supranuclear vacuoles’ disappearance, epithelial cell fold fusion, inflammatory cell infiltration into lamina propria, and thickening of lamina propria and submucosa (4). Feed-induced abnormal conditions could weaken the vital immune defense functions in the intestine. Therefore, intestinal health status can be one of the defining factors of fish welfare.

Mammalian studies have demonstrated that exogenous immunomodulatory feed components that are intended to improve gut health aid in overcoming feed-induced enteropathy by preventing the destruction of epithelial cells as well as an increase of para- and trans-cellular permeability (5). Probiotics can improve the intestinal epithelial barrier function by stimulating and stabilizing the gut mucosal immunological barrier (6). They have been found to affect the growth of beneficial microorganisms positively and alleviate ulcerative colitis in human beings (7). Experimental murine models that were used to study colitis also revealed the anti-inflammatory properties and proinflammatory cytokine activity blocking function of probiotics (8).

The application of probiotics as an alternative disease management strategy for farmed fish is documented in several reviews (9–11). Although probiotic bacterial administration (lactic acid and Bacillus types) is a promising approach to maintaining the intestinal health of fish, only some studies have assessed this aspect (12–14). The ability of microalga (*Chlorella vulgaris*) and yeast (*Candida utilis*) in maintaining intestinal homeostasis and combating soybean-induced enteropathy has been described in Atlantic salmon (15).

Intestinal inflammation in fish has been studied using chemically induced experimental models. In zebrafish, epithelial damage, goblet cell depletion, granulocyte influx, and increased expression of interleukin-1beta, tumor necrosis factor-alpha, and interleukin-10 were the characteristics of oxazolone-induced inflammation (16). In the present study, it was hypothesized that a microbial feed additive (a lactic acid bacterium – *Pediococcus acidilactici*) could alleviate the oxazolone-induced inflammatory responses in Atlantic salmon. The aim of the study was to find the differences in immune responses between groups of fish, which were fed a microbial additive-coated or phosphate-buffered saline (PBS)-coated feeds, at different time points after inflammation. The ability of a commercially available microbial feed additive in countering the induced inflammation will be discussed based on the alterations in micromorphological features, selected inflammation-related genes, and expression of proteins in the distal intestine (DI) of Atlantic salmon.

## Materials and Methods

### Fish

Atlantic salmon smolts (Aquagen strain; av. wt. 150 g) obtained from Cermaq, Hopen, Bodø, Norway and maintained at the Research Station, University of Nordland (UiN), Norway were used for the study. A feeding trial was designed with two fish groups – the test group (Probiotic) was offered microbial feed additive-coated feed, and the control group (Control) received feed without the additive. Twenty fishes were randomly distributed into each of the triplicate tanks (500 l) of the two groups. The water temperature in the flow-through rearing system was 7.5°C, and the oxygen saturation was 89%–95%. The study was approved by the Norwegian Animal Research Authority (FDU: Forsøksdyrutvalget ID 4353), and the handling of fish during intubation as well as sampling procedures were according to the authorized protocols.

### Experimental feeds, feeding

Bactocell^®^ (lyophilized live lactic acid producing bacterial strain *P. acidilactici* CNCM MA18/5M, Lallemand Animal Nutrition, Balgnac, France) was included in the feed for the probiotic group, at the recommended rate of 1 × 10^10^ CFU/kg feed. Briefly, 1 g of Bactocell^®^ was suspended in 100 ml of sterile PBS (pH 7.5), and the solution was stirred overnight. On the following day, the bacterial suspension was spray-coated on 1 kg of commercial feed (Spirit, size: 3 mm, Skretting, Norway) that was being mixed thoroughly to ensure uniform coating. The microbial additive-coated feed was then dried at 30°C for 4 h, cooled at room temperature for 30 min, sealed and stored at 4°C in plastic bags until further use. The control feed was prepared similarly, with the exception that the feed was spray-coated with sterile PBS without the Bactocell^®^. Fresh batches of feeds were prepared and stored every week.

Fish in triplicate tanks of the control and probiotic groups received their respective feeds for a period of three weeks (initial feeding), prior to carrying out the intubation, and for an additional 3 weeks starting from 48 h after the intubation. The feeds were dispensed from a programed automatic feeder (Arvo-Tec, Huutokoski, Finland) at a rate of 1.5% of body weight per day.

### Induction of intestinal inflammation

At the end of the first 3-week feeding period, a chemical allergen, oxazolone (4-ethoxymethylene-2-phenloxazol-5-one; Sigma-Aldrich, St. Louis, USA) was anally intubated to induce inflammation. The fishes were first starved for 48 h and then anesthetized using MS-222 (80 mg/l – Tricaine methanesulphonate, Argent Chemical Laboratories, Redmond, USA). Briefly, 0.5% oxazolone in 50% ethanol was used for the anal intubation – each fish of average weight 150 g received 1 mg of oxazolone in 200 μl of the inoculum. This intubation dosage was determined based on similar studies on zebrafish (16).

In order to deliver the pre-determined volume of allergen into the DI, a veterinary sterile Buster cat catheter (1.3 mm × 130 mm, Kruuse Norge AS, Drøbak, Norway) fitted to a sterile 1 ml syringe was inserted into the anal pore. Following this procedure, and after ensuring the successful delivery of the allergen, the fish were allowed to recover in separate tanks before returning them to their rearing tanks.

### Sampling

A total of nine fish from each group (three from each triplicate tank) were sampled at each time point. The initial samples were taken at the end of the first feeding term of 3 weeks, i.e., ahead of the intubation. Following induction of inflammation, samples were collected at 4 and 24 h, and later after 3 weeks.

Fishes were anesthetized using MS-222 and euthanized before collecting the samples. After drawing out blood, the DI was dissected out. The anterior portion (5 mm) of DI was fixed for the histology study. The remaining segment intended for gene expression and proteomic studies was gently flushed with sterile PBS to remove the digesta, and placed in microtubes that were snap-frozen in liquid nitrogen and stored at −80°C.

### Histological study

Approximately 5 mm of the distal intestinal samples (*n* = 6) were rinsed with PBS and fixed in 4% phosphate-buffered formaldehyde solution (pH 7.2) at 4°C for 24 h. Standard histological procedures were adopted for dehydration, processing, and paraffin embedding. The paraffin blocks thus prepared were sectioned using a microtome (Microm HM3555, MICROM International GmbH, Walldorf, Germany). Five-micrometer thick longitudinal sections were cut and mounted on SuperFrost^®^ slides (Menzel, Braunschweig, Germany), and a robot slide stainer (Microm HMS 760×, MICROM International GmbH) was used to stain the slides with Alcian Blue-Periodic Acid Schiff’s reagent (AB-PAS, pH 2.5). First, all acid mucins were stained blue with alcian blue, and in the subsequent PAS reaction only the neutral mucins were stained magenta, as described by Bancroft and Gamble (17). Photomicrographs were prepared using light microscopy employing Olympus BX61/Camera Color View *III*u (Olympus Europa GmbH, Hamburg, Germany) and photo program Cell P (Soft Imaging System GmbH, Munster, Germany).

### Gene expression study

The mRNA levels of selected genes, namely, mitochondrial ubiquitin ligase activator of NFκB1 (*mul1b*), interleukin 1b (*il1b*), tumor necrosis factor a (*tnfa*), interferon gamma (*ifng*), acute-phase serum amyloid A-5 protein (*saa5*), interleukin-10 (*il10*), annexin A1 (*anxa1*), and immunoglobulin T (*igt*) were assessed in this study.

#### RNA Extraction and cDNA Synthesis

Total RNA from the frozen tissue was isolated using acidic phenol chloroform extraction and alcohol precipitation method (18). RNA quantity was measured by Quant-iT^™^ RNA broad range assay kit (Invitrogen, Carlsbad, USA) and Qubit^®^ 2.0 Fluorometer (Life Technologies, Carlsbad, USA), and its integrity was confirmed by 1% agarose gel electrophoresis. Reverse transcription was carried out using QuantiTect reverse transcription kit (Qiagen GmbH, Hilden, Germany) with 1000 ng of total RNA in a 20 μl reaction volume, as mentioned in the manufacturer’s protocol. The cDNA obtained were subjected to 10-fold dilution, before being used in qPCR.

#### Real-Time PCR (qPCR) and Quantification of Gene Expression

The qPCR was performed on StepOnePlus™ Real-Time PCR System (Applied Biosystems, Carlsbad, USA) as described by Lokesh et al. (19). Fast SYBR^®^ Green PCR Master Mix (Applied Biosystems) was used for all reactions. Reactions of 10 μl total volume consisted of 5 μl of Fast SYBR^®^ Green PCR Master Mix, mixed with 1 μl primer mix (200 nM), 2 μl cDNA (5 ng/μl) and 2 μl of nuclease free water. Thermal cycling conditions were: initial holding at 95°C for 20 s followed by 40 cycles of denaturation at 95°C (3 s), and annealing/extension at 60°C (30 s). A melt curve analysis for each sample was performed to check the specificity of the primers. Reactions were performed in duplicate on individual fish samples (*n* = 9). A relative standard curve method was employed to calculate the gene expression. The standard curve was obtained by running a 6-point threefold dilution series on pooled total RNA from all the samples normalized to 1000 ng. The dilution series was reverse transcribed and used for qPCR. The efficiency of the primers was calculated using the equation *E* = (10^−1/m^− 1) 100. Using geNorm (20), the normalization factor was computed for each of the samples based on the relative quantities of the two most stable genes (*ef1ab* and *rnap2*) from among the set of four reference genes – elongation factor 1AB (*ef1ab*), RNA polymerase II (*rnap2*), hypoxanthine phosphoribosyltransferase 1 (*hprt1*), and ubiquitin (*ubi*). The expression levels of all the target genes were then calculated relative to the normalization factor (21). The primers for the reference and target genes used in the study are given in Table 1.

**Table 1:** **List of primers used in the present study**.

Gene	Primer sequence	PCR efficiency (%)	Amplicon size (bp)	Reference
*ef1ab*	TGCCCCTCCAGGATGTCTAC F CACGGCCCACAGGTACTG R	98.6	59	GenBank: BG933853
*rnap 2*	CCAATACATGACCAAATATGAAAGG F ATGATGATGGGGATCTTCCTGC R	96.82	157	GenBank: BG936649
*hprt1*	CCGCCTCAAGAGCTACTGTAAT F GTCTGGAACCTCAAACCCTATG R	99.87	255	GenBank: BT043501
*ubi*	AGCTGGCCCAGAAGTACAACTGTG F CCACAAAAAGCACCAAGCCAAC R	97	162	GenBank: AB036060.1
*il1b*	GCTGGAGAGTGCTGTGGAAGA F TGCTTCCCTCCTGCTCGTAG R	102.9	73	GenBank: AY617117
*tnfa*	TGCTGGCAATGCAAAAGTAG F AGCCTGGCTGTAAACGAAGA R	105.5	178	GenBank: AY848945
*il10*	CGCTATGGACAGCATCCT F AAGTGGTTGTTCTGCGTT R	102.1	80	GenBank: EF165028
*mul1b*	CCAGAACGACCAACAGGAAGG F GTGAACTCTCTCCAGGAACCAGC R	94.1	137	GenBank: JF933931
*ifng*	CTAAAGAAGGACAACCGCAG F CACCGTTAGAGGGAGAAATG R	97.4	159	GenBank: AY795563
*saa5*	GCAGCAGCAGTCATCAGTA F AGTTCCTTGGGAGTCCATTT R	97.8	151	GenBank: NM_001146565.1
*igt*	CAACACTGACTGGAACAACAAGGT F CGTCAGCGGTTCTGTTTTGGA R	96.4	97	GenBank: GQ907004
*anxa1*	GTCAGAATCTTGGTCCTGGTTC F ACTGCCGTAGTGAAGTGTGCT R	98.7	98	GenBank: CA060324

### Protein expression study

Protein expression study was performed on the samples procured at 24 h after inducing inflammation. Protein samples were extracted from DI collected from the two groups (*n* = 6) to perform 2-dimensional gel electrophoresis (2-DE).

#### Protein Extraction and Two-Dimensional Electrophoresis

The extraction of proteins was performed as described by Kulkarni et al. (22), with slight modifications. In brief, the frozen intestinal samples (~1 g) were ground to a fine powder, and resuspended in 2 ml of sterile PBS containing 0.1% protease inhibitor cocktail (Sigma). The resulting slurry was subjected to sonication using a vibrating probe (Vibra-Cell VC 750, Sonics and Materials Inc., Newtown, USA) for 30 s with a pulse mode of 10 s on ice, and centrifuged (3000 × *g*, 30 min, 4°C) to obtain crude protein extract as the supernatant. Next, a mix of trichloroacetic acid (TCA, 10% w/v, Sigma) and dithiothreitol (DTT, 0.1%, Sigma) was added to the crude protein extracts and incubated on ice for 30 min to obtain a precipitate. After that, a step of centrifugation (10,000 × *g*, 30 min, 4°C) was performed to pelletize the precipitate, which was then resuspended in cold acetone containing 0.1% DTT (Sigma). Employing a vortex mixer this suspension was mixed intermittently for 30 min at 20°C. A last round of centrifugation (10,000 × *g*, 30 min, 4°C) yielded purified protein pellets that were dissolved in rehydration buffer [9.8M urea (Bio-Rad, Hercules, USA), 2% CHAPS (Sigma), 20 mM DTT (Sigma), 0.5% BioLyte 3-10 (Bio-Rad) and 0.001% bromophenol blue (Sigma)]. A fraction of the solubilized protein was dialyzed using 3 kDa cut off Nanosep spin columns (VWR International, Oslo, Norway). The quantitation of the dialyzed protein was performed using Qubit^®^ Protein Assay Kit and Qubit^®^ Fluorometer (Life Technologies).

For the first dimension, 100 μg (300 μl) of protein from the aforementioned step was used to rehydrate 17 cm immobilized pH gradient (IPG) gel strips pH 3-10 (Bio-Rad) as per the manufacturer’s instructions. Subsequent isoelectric focusing (IEF) was performed on the rehydrated IPG strips using the preset method within the Protean IEF cell (Bio-Rad), i.e., a maximum of 10,000 V was subjected to the IPG strips in 3 slow ramping steps ultimately reaching a total of 60,000 vh at a constant temperature of 20°C. The electro-focused IPG strips were first reduced and then alkylated for 15 min each in equilibration buffer (6M urea, 0.375 M Tris-HCl, pH 8.8, 2% SDS, 20% glycerol) containing 0.2% DTT followed by 0.3% iodoacetamide (Bio-Rad), respectively. The second dimension gel electrophoresis of the equilibrated strips was performed on a 12.5% polyacrylamide gel in PROTEAN II xi system (Bio-Rad) followed by staining with Sypro^®^ Ruby protein gel stain (Life Technologies). Further, the gel images were captured using ChemiDoc™ XRS imaging system (Bio-Rad).

#### Gel Image Analyses

The gel image analyses that include spot detection, normalization, spot matching, and differential spot volume detection were performed using the PDQuest Advanced software (Bio-Rad). The differentially expressed spots in the two groups are those having a minimum 1.5-fold difference in volumes and statistical significance of *p* < 0.05 by two-tailed Student’s *t-*test. The volumes of the differentially expressed spots were exported to GraphPad Prism v5.04 (GraphPad Software Inc., La Jolla, USA) to further confirm the statistically significant differences, after checking the assumptions of the *t*-test. Transformations were done wherever necessary, and Mann–Whitney test was employed for the analysis of non-parametric data.

#### Protein Identification

The selected protein spots were excised from the preparative Sypro^®^ Ruby stained gel loaded with 300 μg of the protein to carry out liquid chromatography and tandem mass spectrometry (LC-MS/MS). The subsequent in-gel reduction, alkylation followed by trypsinization and the LC-MS/MS analyses (ESI Quad TOF; Micromass/Waters, Milford, USA), were performed at the Tromsø University Proteomics Platform, Norway. The resulting data of LC-MS/MS analyses obtained as the peak list files using Protein Lynx Global Server Software (version 2.1, Micromass/Waters) were used to determine the protein identities; using the Mascot search engine at UiN, Norway. The search was done in the “Actinopterygii (ray-finned fishes)” database. The parameters for the search included one missed cleavage by trypsin, peptide mass tolerance of 100 ppm, 0.1 Da of fragment mass tolerance, carbamidomethyl (of cysteine) for fixed modification, oxidation (of methionine) for variable modifications, and 1+, 2+, and 3+ for the precursor peptide charge state. The protein inference was based on two unique peptides.

### Statistical analyses

The relative mRNA levels of genes were analyzed using the software GraphPad Prism version 5.04. The data before inducing the inflammation is not used for statistical analyses since it introduces an additional factor to the current two-factor model, and the study focuses on the effect of the microbial additive on the induced inflammatory condition. Two-way ANOVA was used to find the interaction between the factors (treatment × time point), and the effect of the two factors. Bonferroni multiple comparisons were performed to understand the difference between two study groups at a particular time point and to find the changes between two time points for a particular group. All the assumptions were checked before performing the tests. Transformations were done wherever necessary. If the data were found to be non-parametric, Kruskal–Wallis and Dunn’s multiple comparison tests were used. The differences were considered significant at *p* < 0.05.

## Results

### Histological observations

**Figure 1:**
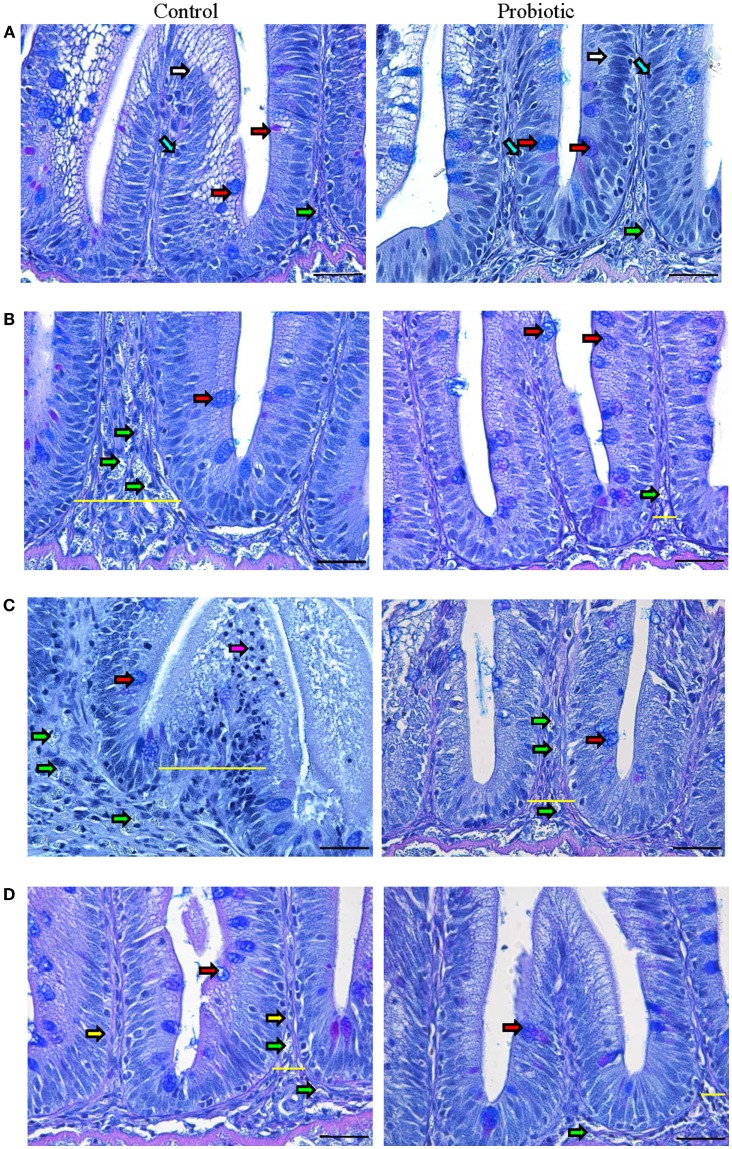
**Photomicrographs of the distal intestine of Atlantic salmon**. The images show the micrographs of distal intestine of control and probiotic groups (*n* = 6 fish) before inducing inflammation, initial **(A)** and at 4 h **(B)**, 24 h **(C)**, and 3 weeks **(D)** after the induction of inflammation. PAS positive acid and neutral mucus-filled goblet cells (red arrow), enterocytes (white arrow), intraepithelial lymphocytes (blue arrow), granulocytes (green arrow), lymphocytes (pink arrow) and lamina propria (yellow arrow) are shown in the figure. The inflammatory condition is discernable at 24 h in the control group, characterized by enlarged intestinal villi and lamina propria (indicated by yellow lines) as well as the appearance of numerous granulocytes. Scale bar: 20 μm.

Normal villi contour, distinct lamina propria, enterocytes with their basal nuclei, and goblet cells were visible in both groups before inducing inflammation (Figure 1A). In the probiotic group, there were more goblet cells, intraepithelial lymphocytes (IELs), and supranuclear vacuoles (Figure S2 in Supplementary Material) in the villi, and more immune cells were evident in the lamina propria.

The onset of inflammation was discernible in the control group at 4 h after the induction of inflammation (Figure 1B; Figure S2 in Supplementary Material), characterized by granulocyte-(neutrophil) infiltration into the widened lamina propria. On the other hand, the probiotic group had more mucus-secreting goblet cells and limited infiltration of granulocytes. By 24 h, the control group was marked by severe edema, widened intestinal villi and lamina propria, dislocated enterocytes and goblet cells, and infiltration of granulocytes and lymphocytes (Figure 1C; Figure S2 in Supplementary Material). In the probiotic group, the inflammatory process became visible only at 24 h, notably due to the presence of granulocytes in the lamina propria and enlargement of intestinal villi. The speed of recovery was also different in the two groups – after 3 weeks, the control group still had wider lamina propria and numerous granulocytes compared to the probiotic group (Figure 1D; Figure S2 in Supplementary Material).

### Gene expression

**Figure 2:**
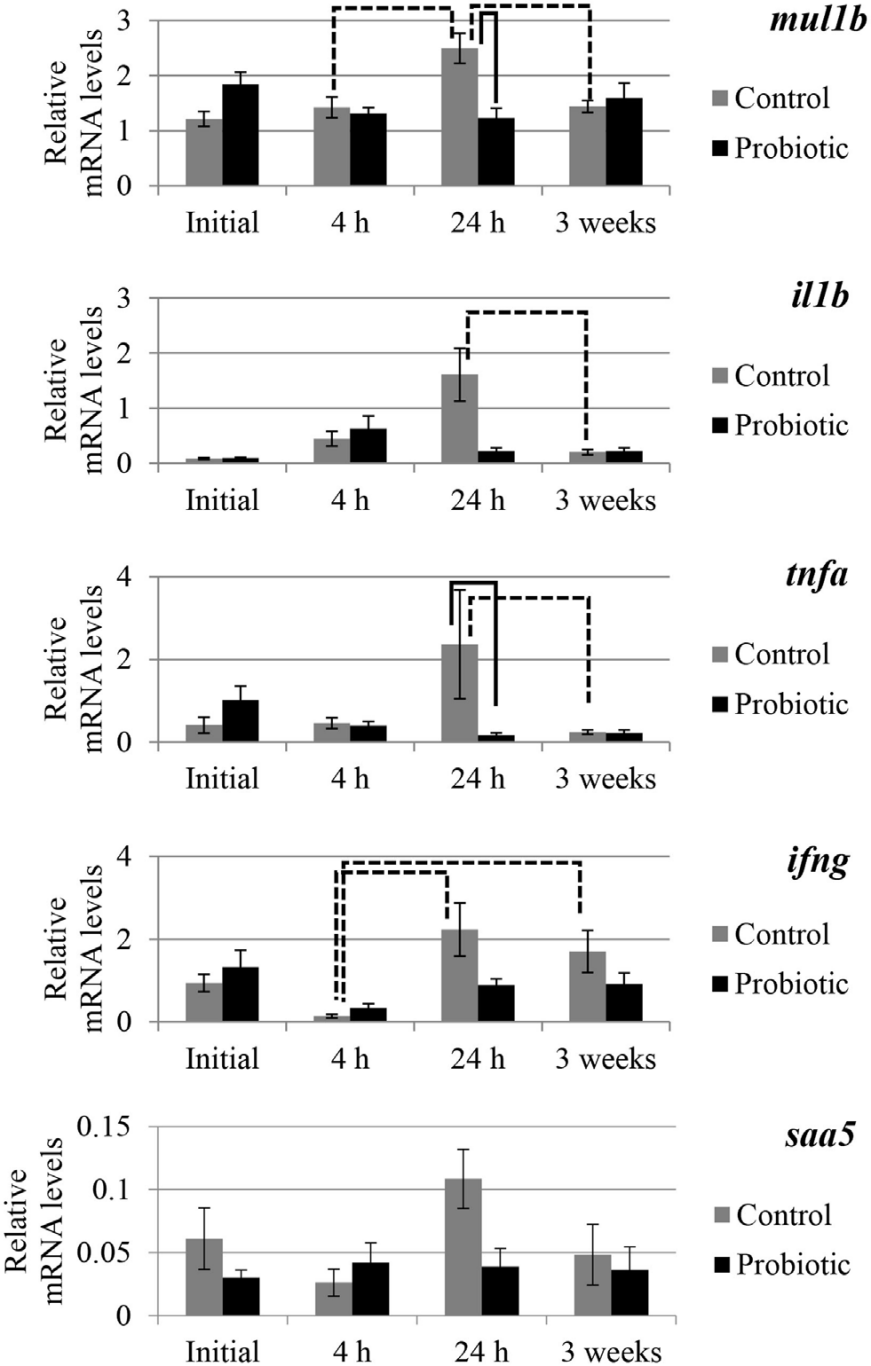
**Relative mRNA levels of *mul1b*, *il1b*, *tnfa*, *ifng*, and *saa5* in the distal intestine of Atlantic salmon**. The distal intestinal gene expression of control and probiotic groups (*n* = 9 fish) before inducing inflammation (initial) and at 4 h, 24 h, and 3 weeks after the induction of inflammation are shown in the figure. Solid connectors indicate significant differences (*p* < 0.05) between the study groups at a particular time point. Dashed connectors indicate significant difference (*p* < 0.05) between the levels at two time points of a particular study group. Values are presented as mean ± SEM.

**Figure 3:**
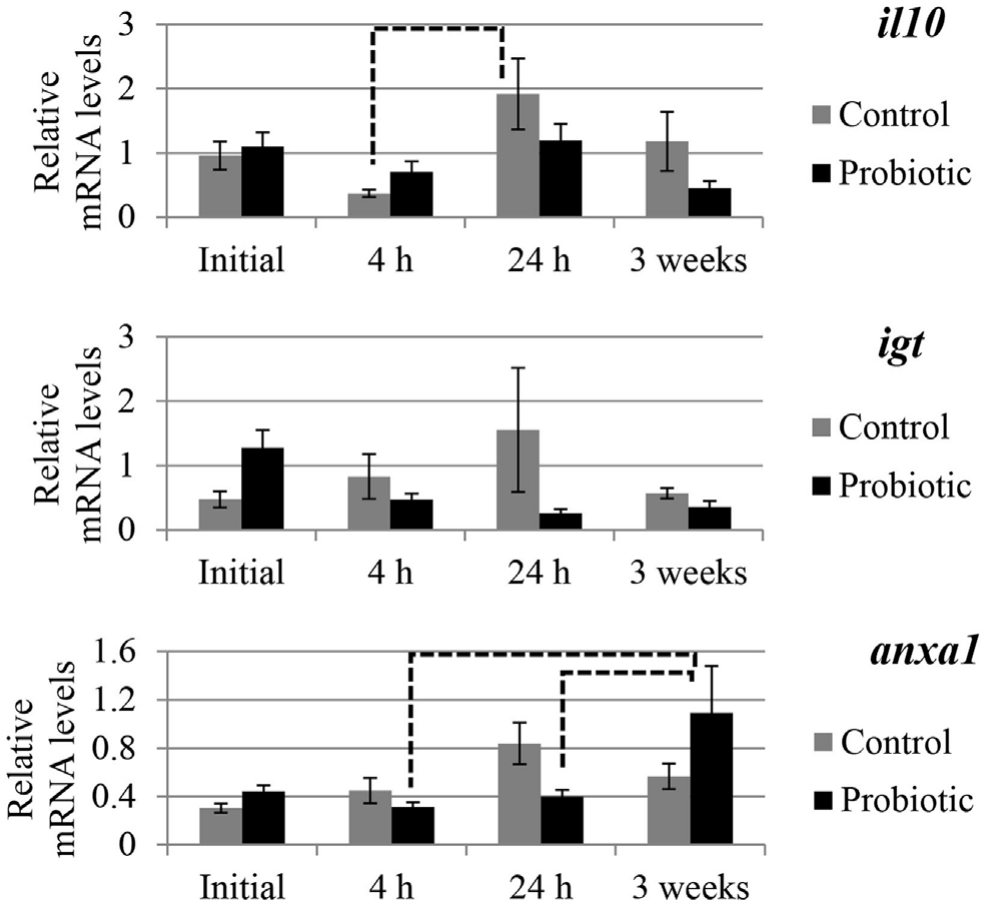
**Relative mRNA levels of *il10*, *igt*, and *anxa1* in the distal intestine of Atlantic salmon**. The distal intestinal gene expression of control and probiotic groups (*n* = 9 fish) before inducing inflammation (initial) and at 4 h, 24 h, and 3 weeks after the induction of inflammation are shown in the figure. Solid connectors indicate significant differences (*p* < 0.05) between the study groups at a particular time point. Dashed connectors indicate significant difference (*p* < 0.05) between the levels at two time points of a particular study group. Values are presented as mean ± SEM.

The expression of selected genes associated with immune and inflammation etiology in the two study groups is shown in Figures 2 and 3. An interaction of the two factors (treatment × time point) was detected for *anxa1*, *mul1b*, and *tnfa*. In general, prominent differences in the expression of the genes between the probiotic and the control groups were seen at 24 h after the induction of inflammation. For all the genes, the mRNA levels in the control fish were greater than those of the probiotic fed fish; the differences being significant in the case of *mul1b* and *tnfa* (Figure 2). Further, the expression of the different genes in the control fish at 24 h was greater compared to the expression at 4 h post-inflammation; significant differences were noted for *mul1b*, *ifng*, and *il10* (Figures 2 and 3). In addition, the control fish had lower levels of genes at 3 weeks compared to the levels at 24 h; significant differences were detected for *mul1b*, *il1b*, and *tnfa* (Figure 2). Further, the level of *anxa1* at 3 weeks is higher in the probiotic group compared to the levels at 4 and 24 h (Figure 3).

### Protein expression

**Figure 4:**
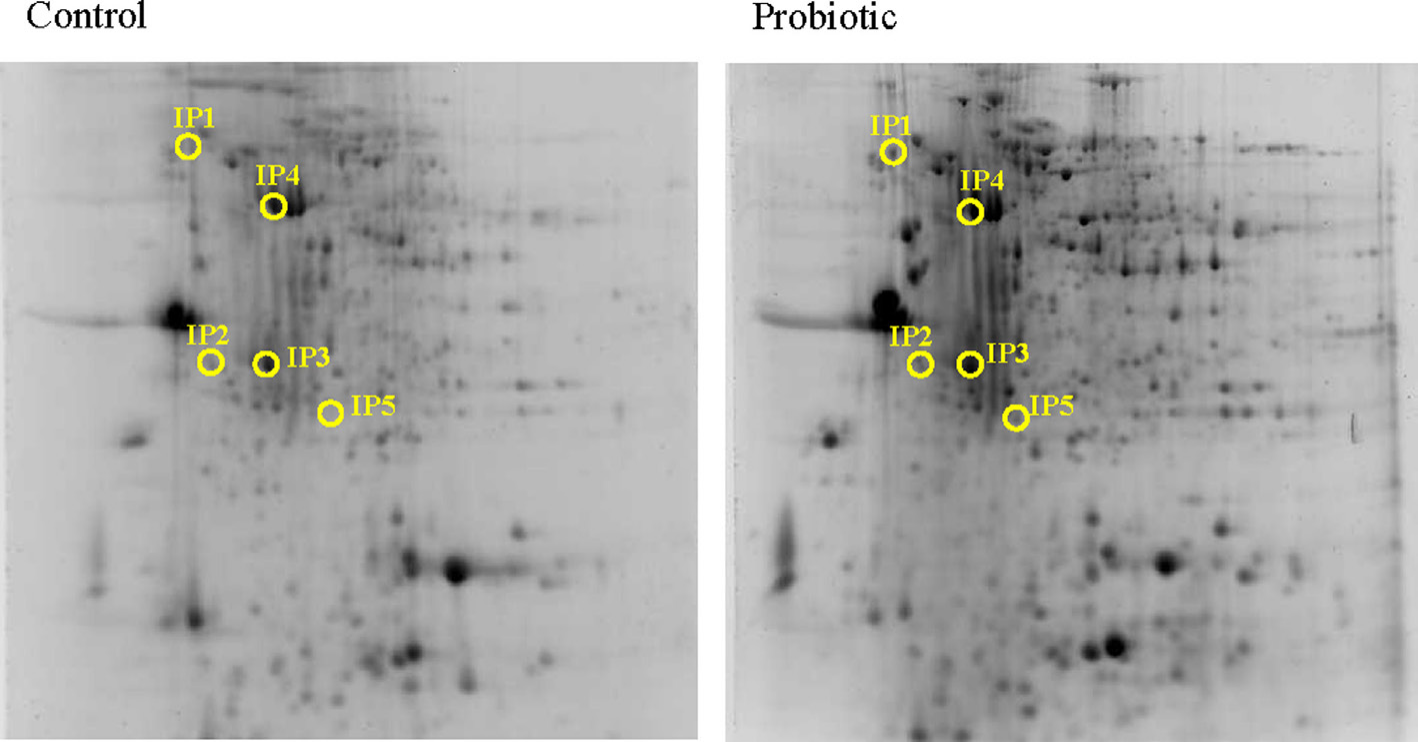
**Representative 2-DE gels generated using protein samples from the distal intestine of Atlantic salmon**. The protein spots in the control and probiotic groups (*n* = 6 fish) at 24 h after the induction of inflammation are shown in the figure. Intestinal proteins from the fish were isoelectrically focused on 17 cm IPG strips (pI 3-10) and were subjected to 12.5% SDS-PAGE. The 2-DE gels were stained with Sypro^®^Ruby protein gel stain, and the spots were annotated using the data from LC-MSMS. The spot numbers in the gels correspond to the protein identities mentioned in Table 2.

Based on the alteration of the selected genes, the protein expression was assessed only at 24 h after inducing inflammation. From among the many protein spots resolved in the gels, the differentially expressed spots corresponded to five inferred proteins. Calreticulin (1.58-fold, Calr), Proteasome subunit alpha type-5 (2.96-fold, Psma5) and Trypsin-1 (3.91-fold, Trp1) were overexpressed at 24 h after inducing inflammation, in the DI of Atlantic salmon that were on microbial additive-coated feed compared to those in the control group (Figure 4 and Tables 2 and 3). On the other hand, Cathepsin B (0.51-fold, Ctsb) and alpha-*N*-acetylgalactosaminidase (0.49-fold, Naga), were the underexpressed proteins.

**Table 2:** **Information on the proteins that were altered significantly in Atlantic salmon intestine**.

Spot no.	Protein accession number and details	Apparent pI/MW (kDa)	Protein score	ST^a^	Mp/Up^b^	SU^c^	Peptide sequence^d^
IP1	Ssa.34432, Clone ssal-rgf-517-369 Calreticulin precursor putative	3.5/86.5	252	54	3/3	252	**FYGDAEADK EAEEFGNETWGTTK FYGDAEADKGLQTSQDAR**
IP2	NP_001134432, Proteasome subunit alpha type-5 [*Salmo salar*]	4.4/33.2	108	43	2/2	108	**IVEIDTHIGCAMSGLIADAK TFHMYSKEELEDVIK**
IP3	Ssa.7877, Transcribed locus, strongly similar to NP_001117776.1 procathepsin B precursor [*Oncorhynchus mykiss*]	5.7/12.3	817	54	8/8	817	**EQQIMSELYK CVSECNAGYTPSYVK GKDECGIESEIVAGIPR TGVYQHVTGQMLGGHAIK NGPVEAAFSVYEDFLLYK DGPVEAAFSVYEDFLLYK DQGSCGSCWAFGAAEAISDR ENDTPYWLVANSWNTDWGDNGFFK**
IP4	Ssa.8176, nagab Alpha-*N*-acetylgalactosaminidase	5.3/33.0	1430	54	16/16	1430	**ASALVFFSR NCISEVLFR ITIDAQTFADWK SGIEVFWRPLSDK FRCDIDCQNDPK VAIGINQDPMGVQGR FPSGIPNLASYIHDR YQASLGQLNYTTGSYK RFPSGIPNLASYIHDR ELGYVYVNIDDCWASK SQMALWAIMAAPLFMSNDLR LGIYGDMGTLTCGGYPGTPLDK WNDPDMLVVGDFGLSMDQSR SQMALWAIMAAPLFMSNDLR WNDPDMLVVGDFGLSMDQSR LGIYGDMGTLTCGGYPGTPLDK**
IP5	Ssa.628, trp-ia Trypsin IA	5.3/61.3	256	54	3/3	256	**VTEGSEQFISSSR HPNYSSYNIDNDIMLIK LGEHNIQVTEGSEQFISSSR**

*^a^Significant threshold score*.

*^b^Total matched peptides/total unique peptides*.

*^c^Total score of unique peptides*.

*^d^Unique peptide sequences are in bold*.

**Table 3:** **Expressional changes of the identified proteins in the distal intestine of Atlantic salmon**.

Spot no.	Protein name	Fold change
IP1	Calreticulin precursor, Calr	1.58 ↑
IP2	Proteasome subunit alpha type-5, Psma5	2.96 ↑
IP3	Cathepsin B precursor, Catsb	0.51 ↓
IP4	Alpha-*N*-acetylgalactosaminidase, Naga	0.49 ↓
IP5	Trypsin-1A precursor, Trp1	3.91 ↑

*↑ indicates overexpression and ↓ indicates underexpression*.

## Discussion

Probiotics promote gut epithelial homeostasis by controlling the altered commensal bacterial composition, preventing pathogenic bacterial invasion, enhancing intestinal epithelial barrier function, and maintaining a balance between Th1/Th2 and Treg cells (23–25). The present study has obtained interesting findings in fish to support the beneficial properties of probiotics that are well-documented in the case of terrestrial animals (26). In human beings, probiotics have been used to treat intestinal infection and inflammation (27, 28), and in this study, the ability of a microbial feed additive to suppress/alleviate the inflammatory responses in the distal intestinal segment of Atlantic salmon was assessed.

### Intestinal micromorphology indicates that the microbial feed additive may have a protective function

The cardinal characteristics of an inflamed tissue include tissue damage, infiltration of lymphocytes/neutrophils, and tight junction damage that increases cellular permeability (29, 30). Tissue inflammation occurs when the mucosal barrier integrity is disrupted, causing increased uptake of luminal antigens to activate T cells, which is followed by the release of a number of inflammatory mediators such as cytokines and chemokines (TNFα and interleukins) – this causes the further recruitment of T cells and upregulation of adhesion molecules, which leads to homing of neutrophils/lymphocytes from blood to inflamed tissues (31). In the present study, the influx of inflammatory cells and disturbance of normal intestinal structure occurred in the control group by 4 h after the induction of inflammation. However, such changes were not evident at this time point in the probiotic group that had more goblet cells and IELs. An earlier study on red tilapia (*Oreochromis niloticus*) has also reported the abundance of IELs in the posterior intestine of the fish upon *P. acidilactici*-feeding (13). Another study on the same fish species that used *Bacillus amyloliquefaciens* as probiotic reported more mucus-secreting cells and IELs (12). The control group had many inflammatory cells, and greater damage of intestinal cells at 24 h compared to the probiotic group. In mammals, acute inflammation is characterized by a large number of neutrophils’ recruitment (within minutes following inflammation stimuli, peaking by 24–48 h) into the lamina propria, with their accompanying macrophages (appearing at the inflammation sites within 2–3 h, and increasing following a time lapse), and lymphocytes (arriving a few days after) (32, 33). The presence of many inflammatory cells in the control group at 24 h may be indicating the severity of inflammation. At this time point, the key inflammatory cytokines (described later) were induced, all pointing to the acute inflammation in the control group. The inflammatory process in the probiotic group, which had less granulocyte-infiltration at 4 h, became visible at 24 h. It is known that reduction in size and number of goblet cells is a characteristic feature of human ulcerative colitis (34). Further, the soluble proteins from *Lactobacillus* GG (LGG) in fermented milk activated epidermal growth factor receptor (EGFR) and anti-apoptotic factor (Akt) in young adult mouse colon cells, and this activation is attributed to the reduction in colitis (35). In the current study, the ability of the microbial additive in preventing inflammatory responses is evident from the subdued inflammation in the probiotic group, based on intestinal micromorphology.

The speed of recovery in the probiotic group was evident since even after 3 weeks the control group had wider lamina propria and numerous granulocytes. Mammalian epithelial turnover occurs every 3–5 days (36), and during epithelial restitution – with the help of antimicrobial peptides, defensins, regenerating protein family – intestinal cells proliferate, expand, migrate, and differentiate, and mucins from goblet cells prevent translocation of commensal bacteria (30). It is reported that in Atlantic salmon, the distal intestinal epithelial turnover occurs after 28 days (37). In mammals, during intestinal wound healing, the denuded area will be rapidly re-sealed by the migration of epithelial cells adjacent to injury, epithelial cell proliferation, maturation, and differentiation (38). In the control fish of the present study, intestinal epithelial cells that were more damaged than those in the probiotic group, were repaired, and reconstructed by 21 days.

### Inflammation associated responses, as evidenced by gene expression

The molecular mapping of genes associated with immune and inflammation etiology aided in comparing the level of inflammation between the two study groups. Upon immune challenge the mRNA of *mul1b*, the activator of NFκB1, is upregulated in Atlantic salmon (39). Effector molecules of the innate immune system play an important role in initiating immune tolerance, intestinal epithelial cell proliferation and tissue repair to maintain intestinal homeostasis, and NFκB, the main mediator, has canonical (proinflammatory) and alternative (anti-inflammatory) pathways (40, 41). The possible NFκB suppression at 24 h (based on *mul1b* downregulation) in probiotic group and the upregulation of the proinflammatory cytokine levels in the control group (*mul1b*, *il1b*, *ifng*, and *tnfa* – time-wise variations; *tnfa* – control vs probiotic) suggest that the immune responses are counteracting the inflammation. The significantly high expression of the key markers of inflammation only in the control group indicates the extremity of inflammation. In a study on tilapia, 3 weeks feeding with *P. acidilactici* upregulated *tnfa* in the mid intestine of the fish (42), whereas in rainbow trout (*Oncorhynchus mykiss*), 3 weeks of *Lactobacillus plantarum* feeding led to the upregulation of intestinal *il8* and not *tlr5* or *igt* (43). In the current study, there was an apparent upregulation of *tnfa* (though not statistically significant) in the probiotic group at the initial time point (after 3 weeks feeding). In mammals, probiotics promote gut epithelial homeostasis by upregulation of IL10 and TGFβ, downregulation of IL12, TNFα, and IL8, and by lowering the NFκB activation in lamina propria mononuclear cells to counter inflammatory responses (23–25, 44, 45). Intake of probiotic, mainly the *Lactobacillus* strain, helped in balancing the pro- and anti-inflammatory cytokines (46), thereby attenuating proinflammatory activity. Administration of *L. plantarum* and *L. brevis* reduced *IL1*β, *TNF*α, and *IFN*γ, as well as the protein expressions of IL1β and IL6, and the signs of colitis in DSS-induced colitic mice (47). The soluble proteins produced by probiotic organisms are found to reduce the TNF-induced epithelial damage and apoptosis in cultured mouse colon explants (48). The microbial feed additive used in this study might have helped to balance the pro- and anti-inflammatory cytokines and maintain intestinal homeostasis in Atlantic salmon.

Elevation of immunoglobulin in response to probiotic feeding has been reported in other studies on animals, including fish (11). Further, tissue IgG1 and IgG2a were lower in the inflammatory regions in mice fed LAB (*Bifidobacterium breve*, *B. bifidum*, and *Lactobacillus acidophilus*) fermented milk than those fed saline and unfermented milk (49). Likewise, at 24 h the levels of *igt* in the intestine of Atlantic salmon fed on probiotic was apparently low, although not significantly. *anxa1* suppresses the inflammatory responses and is known to possess both gastro-protective and anti-inflammatory properties (50), as seen in the repair of mice intestinal mucosal epithelium (51). In Atlantic salmon of the probiotic group, the recovery by the third week from the minor inflammatory responses at 24 h may have been aided, by among others, the overexpression of *anxa1*. The continuous supplementation of the microbial additive, even after inducing inflammation could have positively helped the fish to restore its intestinal integrity.

Although the intestinal micromorphological observations at 4 h point to the differences in inflammatory responses between the control and the probiotic groups, gene expressional differences become visible only when the inflammation in the control group is severe. At 24 h, *mul1b* and *tnfa* in the control group are higher compared to those in the probiotic group. The higher levels of *mul1b* and *ifng* at 24 h compared to the values at 4 h coincide with the severity of inflammation at 24 h, in the control group. In addition, the higher levels of *mul1b*, *il1b*, and *tnfa* at 24 h compared with the levels at 3 weeks also reflect the histological observations. It should be noted that the anti-inflammatory gene, *il10*, was higher only in the control group at 24 h compared to the value at 4 h. On the other hand, even though the inflammation in the probiotic group is not as severe as that in the control group, the gastro-protection linked gene *anxa1* is high at 3 weeks in the probiotic group, indicating the protective mechanisms enabled by the microbial additive.

### Proteins that contribute to protective responses in the intestine

Five proteins were differentially expressed in the DI of Atlantic salmon that were on microbial additive-incorporated feed compared to those in the control group.

Calreticulin, CALR (or calregulin/CRP55/CaBP3/calsequestrin-like protein/endoplasmic reticulum resident protein 60, ERp60) is a multifunctional endoplasmic resident chaperone protein which has also been identified in the cytoplasm, cell membrane, and extracellular matrix. The non-endoplasmic reticulum functions of calreticulin has also been studied (52) – calreticulin-induced cell proliferation and faster healing were demonstrated in murine diabetic wound model. Adhesion of epithelial cells to their neighbors and extracellular matrix is governed by adhesion complexes, and the integrin-interacting CALR is associated with the adhesion complex of the focal junctions (53). Thin calreticulin-containing tubules that encircle mucin granules of goblet cells are in close contact with endoplasmic reticulum tubules (54). The increased presence of the goblet cells and the significant upregulation of Calr in the probiotic group point to the key role of this protein in many of the cellular and immunoregulatory functions, which help to counter the inflammation.

Proteasome subunit alpha type-5 (PSMA5) is a proteinase complex that performs the non-lysosomal ATP/ubiquitin-dependent peptide-cleavage. The α-ring of the proteasome is involved in recognition, binding of substrates as well as their entry into the proteasome chamber (55). The inner rings of the constitutive proteasome complex that contain proteolytic centers are encoded by 3 β-subunits (β1, β2, and β5), which get converted to immuno-subunits upon stimulation by IFNγ, to form immunoproteasome (55, 56). Processing of class I MHC peptides is undertaken by the immunoproteasome, which displays increased chymotrypsin-like activity than its constitutive counterpart (56). However, in the present study, *ifng* was not upregulated and it is not the β-subunit that is overexpressed in the probiotic group at 24 h. Information on immunoproteasome in fish is scanty. Some immune-related proteasome subunits are present in hagfish, *Eptatretus burgeri* – 20S proteasome subunit, a and b type, 1–7 and proteasome activator subunit 3 (57). *psma5*, *psmb3*, and *psmd6* were lower in triploid, and immature diploid rainbow trout, *O. mykiss*, and their expression has been linked to the feed rations (58). The microbial additive could have increased Psma5 to enhance its recognition for further processing of the microbial substrate.

The significant enhancement of Trypsin-1 (Trp1) or cationic trypsinogen in the intestine of the probiotic group at 24 h is an evidence on the homeostatic response in the fish. There are reports that proper administration of trypsin and chymotrypsin effectively reduces inflammation and edema (59). Enterocytes and goblet cells produce enteropeptidase that activates trypsinogen to trypsin (60), and trypsin stored as trypsinogen in paneth cells processes human paneth cell defensins (61). Trypsin was found to localize on mucus-secreting surface epithelial cells of Atlantic salmon, and the protein is suggested to be a part of non-specific immune defense (62). Although the upregulation of trypsin-like activity in the distal intestinal wall was suggested to be associated with subacute enteritis severity (63), Trp1 overexpression was noted in microbial additive-fed salmon that had subdued inflammatory responses. The overexpressed protein may be pointing to the link with intestinal immune defense.

Cathepsin B (CTSB), a lysosomal enzyme belonging to peptidases, helps in intracellular degradation and protein turnover (64). CTSB processes antigens, mainly to activate Th2 cells in mice (65), indicating the immune response regulatory function of this molecule. Upregulation of this enzyme is linked to conditions such as cell death and inflammation (66, 67). Therefore, the underexpression of Ctsb precursor and the mRNA levels of *il1b*, in Atlantic salmon, may have led to the milder inflammatory condition in the probiotic group at 24 h.

Alpha-*N*-acetylgalactosaminidase (NAGA), a glycoside hydrolase, is present in the lumen of human lysosomes (68). This glycosidase helps in the degradation of mucin carbohydrates and removes terminal Alpha-N-acetylgalactosamine residues from glycolipids and glycoproteins (69). When NAGA deglycosylates Gc protein (serum vitamin D3-binding protein) its conversion to the precursor of a principal macrophage-activating factor, MAF is not possible, and immunosuppression will be the net result (70). The underexpression of Naga in Atlantic salmon could be suggesting that probiotic feeding may not cause immunosuppression.

## Conclusion

The findings from the present study provide evidence on the role of the microbial additive in intestinal homeostasis. The milder and delayed inflammatory responses in the probiotic group contrast with the rapid and severe inflammatory pathology in the DI of fish that did not receive the microbial supplement. The speed of recovery was also different in the two groups – the probiotic fed fish overcame the inflammatory challenge rapidly, possibly because of the protective functions that prevailed in this fish.

## Author Contributions

VK designed and led the study. GV, AK, JL, and YK performed the experiments on the fish and were involved in the gene and protein expression studies and interpretation of data. DD conducted histological studies and analyzed the data. VK and GV interpreted the data and wrote the manuscript. All the authors are accountable for the accuracy and integrity of the work and have read, revised, and approved the manuscript.

## Conflict of Interest Statement

The authors declare that they have no competing interests. The research was conducted using the product provided by Lallemand Animal Nutrition, Balgnac, France. There are no commercial or financial relationships with the company that could be considered as a potential conflict of interest. This study was funded by the University of Nordland and the Nordland County, Norway.

## Supplementary Material

The Supplementary Material for this article can be found online at http://journal.frontiersin.org/article/10.3389/fimmu.2015.00409
