# Supplementary figures and images for: A Microbial Feed Additive Abates Intestinal Inflammation in Atlantic Salmon
Source: Front Immunol. 2015 Aug 19;6:409. doi: 10.3389/fimmu.2015.00409 (PMC4541333; doi:10.3389/fimmu.2015.00409)

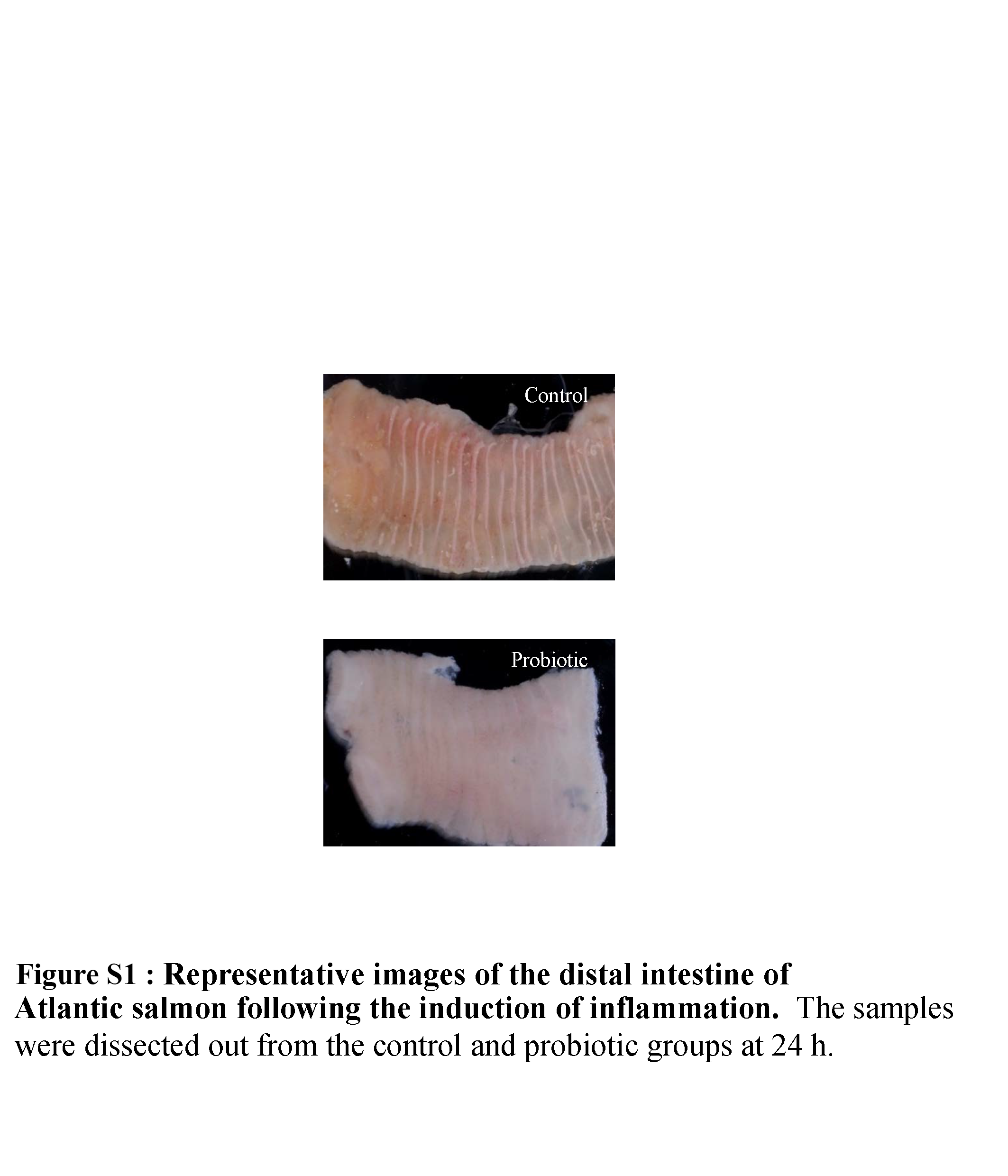

Supplement: Supplementary file 1 [file Image_1.TIF]

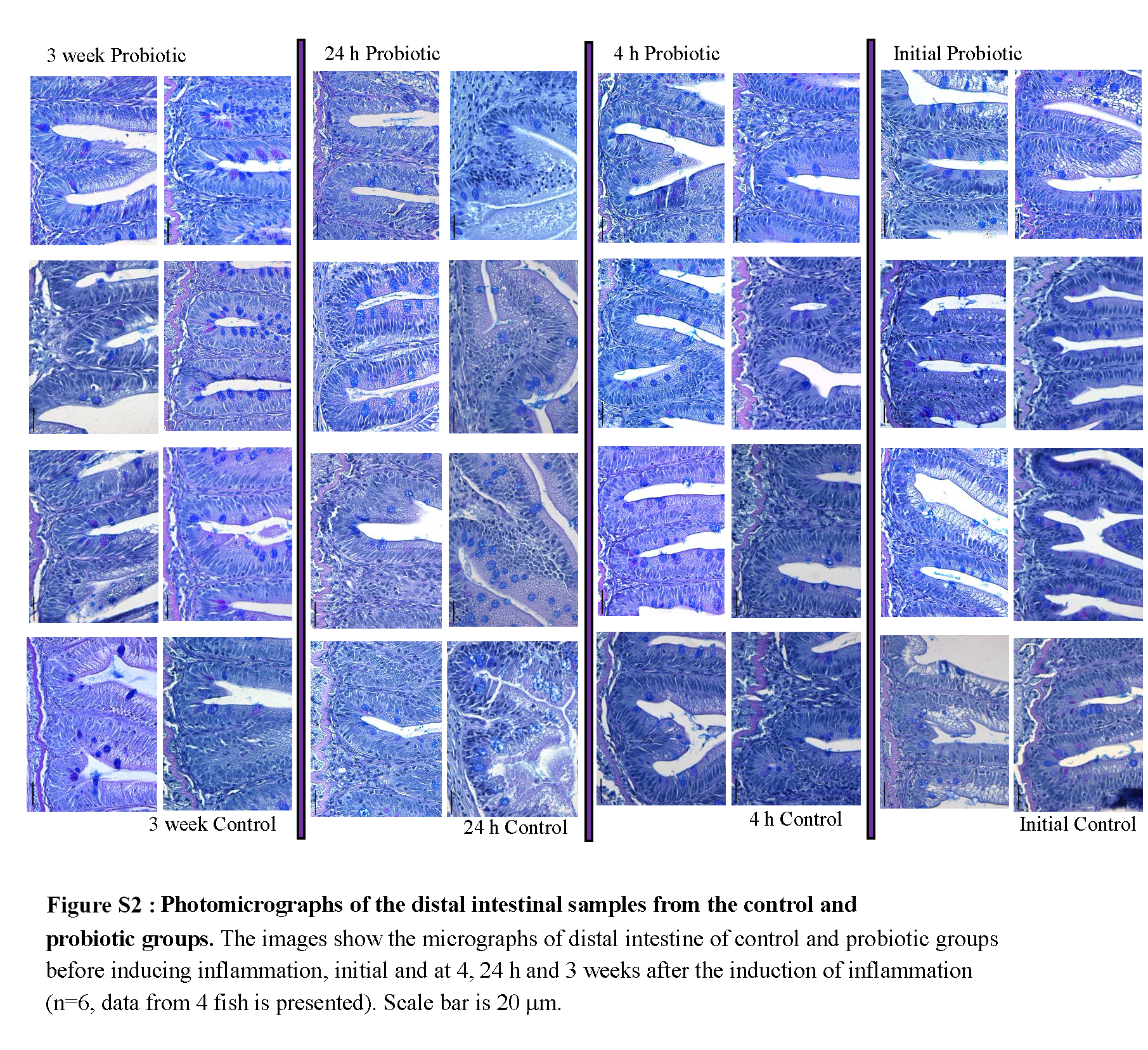

Supplement: Supplementary file 2 [file Image_2.TIF]

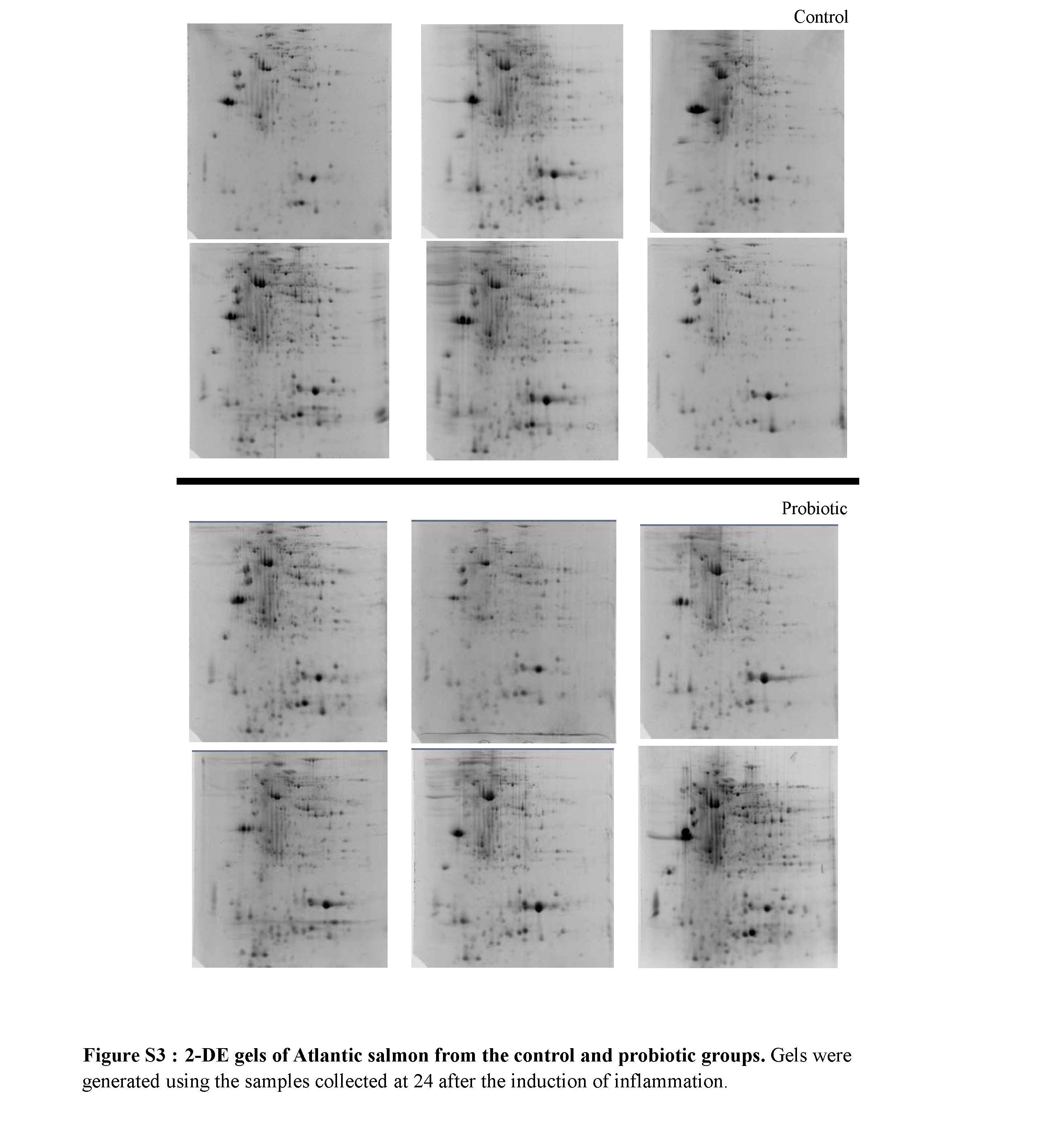

Supplement: Supplementary file 3 [file Image_3.TIF]

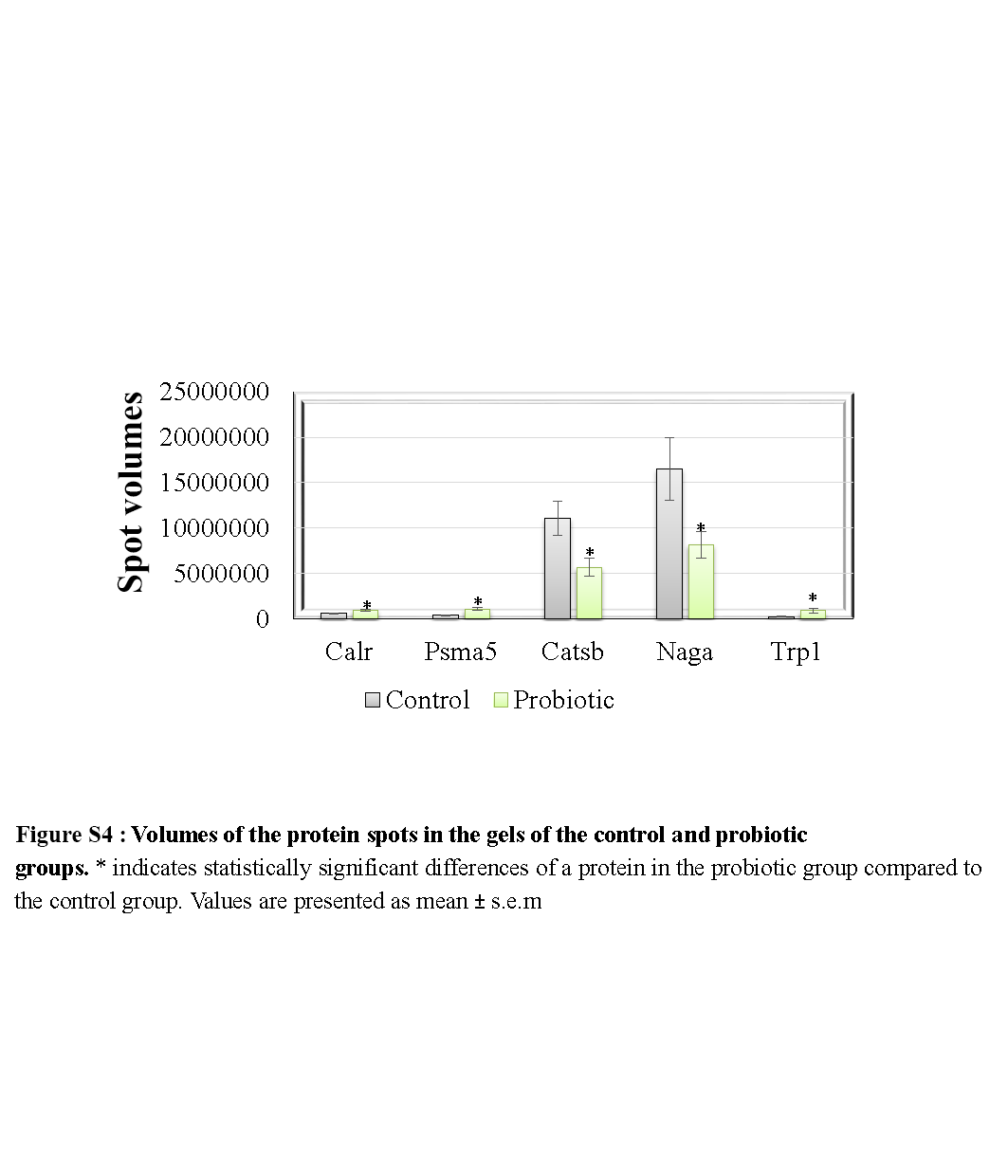

Supplement: Supplementary file 4 [file Image_4.TIF]
